# Complete genome sequence of *Acinetobacter* bacterium strain BZX-2 isolated from hybrid sturgeon

**DOI:** 10.1128/mra.00180-26

**Published:** 2026-07-27

**Authors:** Baodi Shang, Xiaoyi Li, Xiaoping Zhang, Feng Chen, Fei Zhao, Feixiong Chen, Xiaodong Shen

**Affiliations:** 1 Guizhou Academy of Agricultural Sciences, Guizhou Fisheries Research Institute 650994, Guiyang, China; 2 Guizhou Special Aquatic Products Engineering Technology Center, Guiyang, China; University of Southern California, Los Angeles, California, USA

**Keywords:** *Acinetobacter*, hybrid sturgeon, complete genome

## Abstract

We report the complete genome sequence of *Acinetobacter* sp. strain BZX-2, isolated from a hybrid sturgeon (*Acipenser baerii* ♀ × *Acipenser schrenckii* ♂). The genome consists of a 3,819,278-bp chromosome with 3,566 predicted protein-coding genes. The genomic characteristics and antibiotic resistance genes identified provide a basis for the prevention and control of sturgeon diseases.

## ANNOUNCEMENT

*Acinetobacter* are gram-negative, aerobic, non-motile bacteria widely distributed in aquatic and terrestrial environments (1). Members of this genus are widely recognized as opportunistic human pathogens, especially *Acinetobacter baumannii* in nosocomial infections (2, 3). Recent studies have discovered *Acinetobacter lwoffii* in sturgeon with sickness, which induced pathological alterations in several organs and disrupted the balance of gut microbiota (4, 5).

In this study, *Acinetobacter* bacterium strain BZX-2 was isolated from the head surface of a diseased hybrid sturgeon (*Acipenser baerii* ♀ × *Acipenser schrenckii* ♂) collected from sturgeon farms in Huishui (124°48′54.36″E, 40°41′39.48″N), Guizhou Province, China in April 2024. Mucus was scraped from ulcerated lesions on the fish body surface using a sterile scalpel, preserved in cryotubes containing 25% glycerol (vol/vol), and transported to the laboratory. Under aseptic conditions, the mucus was streaked onto brain heart infusion (BHI) agar and incubated at 28°C for 48 h. Pure colonies were obtained by successive sub-cultivation. The strain BZX-2 has been deposited in the Marine Culture Collection of China with no. MCCC 1K10525. No fish were sacrificed, and no lethal or invasive procedures were conducted throughout the experiment.

The strain was streaked from −80°C glycerol stocks onto BHI agar plates and incubated at 28°C for 24 h. Bacterial cells were harvested by centrifugation and submitted to Novogene Co., Ltd. (Beijing, China) for genomic DNA extraction and whole-genome sequencing. Genomic DNA extraction was performed using the STE methodology (6). DNA samples that passed the quality control were used to construct libraries on the PacBio and Illumina sequencing platforms.

SMRTbell libraries were prepared using the SMRTbell Template Kit (version 2.0) and sequenced on the PacBio Sequel II and IIe platforms. PE150 was performed on Illumina NovaSeq. DNA samples were randomly sheared into 350 bp by Covaris sonicator, and DNA libraries were prepared using the NEBNext Ultra DNA Library Prep Kit for Illumina (NEB, USA). The initial draft genome assembly was assembled using Canu v2.0 using the raw sequencing data (7). Canu automatically discovered and removed overlapping termini by matching contig ends to circularize the sequence. Based on the dnaA locus, genome rotation and overlap reduction were performed. The draft contigs were refined three times with long PacBio reads using Racon v1.4.13 and improved genome assembly by three rounds of error correction with short Illumina reads with Pilon v1.22 (8, 9). The Illumina sequencing data provided an average genome coverage of approximately 967×. The assembly yielded a single contiguous sequence.

The genomic analysis of strain BZX-2 was performed using the NCBI Prokaryotic Genome Annotation Pipeline (PGAP) v6.1 (10). Strain BZX-2 contains a single 3,819,278-bp chromosome with 38.74% G + C. The chromosome has 3,566 genes, 80 tRNAs, and 21 rRNAs (Table 1).

**TABLE 1:** Complete genome sequence information of strain BZX-2

Feature	Strain BZX-2
Genome size (bp)	3,819,278
Number of contigs	1 chromosome
GC content	38.74%
Coding sequences	3,566
tRNAs	80
ncRNAs	4
rRNAs	21
Read length (bp)	150
Library layout (paired-end)	2 × 150 bp
PacBio reads	188,905
Reads N50 (bp)	14,361

The genome sequence of strain BZX-2 was compared with those of *Acinetobacter* type strains using CJ Bioscience’s online average nucleotide identity (ANI) calculator and the Type Strain Genome Server to analyze ANI and dDDH values (11, 12), respectively. Strain BZX-2 shared the highest ANI and dDDH values with *Acinetobacter halotolerans* JCM 31009^T^ (GCA_004208515.1) of 92% and 21.3%, respectively.

## Data Availability

The complete genome sequence of *Acinetobacter* sp. strain BZX-2 has been archived in GenBank under accession identifier JBUQDD00000000. The unprocessed sequencing data have been deposited in the NCBI Sequence Read Archive under BioProject accession number PRJNA1419506. The Sequence Read Archive (SRA) accession number is SRR37368766.
